# Peak Finder Metaserver - a novel application for finding peaks in ChIP-seq data

**DOI:** 10.1186/1471-2105-14-280

**Published:** 2013-09-23

**Authors:** Marcin Kruczyk, Husen M Umer, Stefan Enroth, Jan Komorowski

**Affiliations:** 1 Department of Cell and Molecular Biology, Uppsala University, Husargatan 3, Uppsala, Sweden; 2 Postgraduate School of Molecular Medicine, Żwirki i Wigury 61 Street, 02-091, Warszawa, Poland; 3 Department of Immunology, Genetics and Pathology, SciLifeLab Uppsala, Rudbeck Laboratory, Uppsala University, SE-751 85 Uppsala, Sweden; 4 Interdisciplinary Centre for Mathematical and Computational Modelling, University of Warsaw, Pawińskiego 5a Street, 02-106 Warszawa, Poland

**Keywords:** Transcription factor, Peak finder, ChIP-seq, Metaserver

## Abstract

**Background:**

Finding peaks in ChIP-seq is an important process in biological inference. In some cases, such as positioning nucleosomes with specific histone modifications or finding transcription factor binding specificities, the precision of the detected peak plays a significant role. There are several applications for finding peaks (called peak finders) based on different algorithms (e.g. MACS, Erange and HPeak). Benchmark studies have shown that the existing peak finders identify different peaks for the same dataset and it is not known which one is the most accurate. We present the first meta-server called Peak Finder MetaServer (PFMS) that collects results from several peak finders and produces consensus peaks. Our application accepts three standard ChIP-seq data formats: BED, BAM, and SAM.

**Results:**

Sensitivity and specificity of seven widely used peak finders were examined. For the experiments we used three previously studied Transcription Factors (TF) ChIP-seq datasets and identified three of the selected peak finders that returned results with high specificity and very good sensitivity compared to the remaining four. We also ran PFMS using the three selected peak finders on the same TF datasets and achieved higher specificity and sensitivity than the peak finders individually.

**Conclusions:**

We show that combining outputs from up to seven peak finders yields better results than individual peak finders. In addition, three of the seven peak finders outperform the remaining four, and running PFMS with these three returns even more accurate results. Another added value of PFMS is a separate report of the peaks returned by each of the included peak finders.

## Background

The aim of peak finding in ChIP-seq analysis is identification of genomic regions with a high density of mapped sequence tags relative to a measured or estimated background. A simple approach to achieving this goal consists of two steps. Firstly, a sequence of mapped tags along the genome is extracted. Secondly, every contiguous sequence of base pairs with more than a predefined threshold number of tags is selected as an enriched region or binding site. However, the experimental noise and inherent complexities of the tags require more sophisticated algorithms. Numerous solutions have been designed following different statistical models and enrichment measures, including window-based models such as Erange [1], Hidden Markov Model-based methods such as HPeak [2] and others, for example, FindPeaks [3]. Differences in the characteristics of the algorithms result in identification of different sets of peaks for the same ChIP-seq data set. These algorithmic diversities provide an opportunity to analyze ChIP-seq datasets under different conditions, but the problem of deciding which method is optimal for a given data set remains unsolved [4]. Here, in the spirit of protein structure prediction meta-servers, e.g. [5], we present the first meta-solution as a method that combines results from peak-finders chosen by the user.

## Implementation

The implemented meta-server collects results from several peak finders and from these extracts the final result. The peak finders currently included in PFMS are: MACS v1.3.7 [6], CisGenome v2.0 [7], SISSRs v1.4 [8], Erange v2.1 [1], SeqSite v1.0 [9], FindPeaks v3.1.9.2 [3], and HPeak v1.1 [2]. The system can be configured to include any combination of these peak finders. PFMS is implemented in a multi-threading manner. The peak finders can be run in parallel, or sequentially depending on user’s hardware.

### Input

PFMS supports three input data formats: BED, BAM, and SAM. Internally, BAM and SAM formats are converted to the BED format in the initial step of the analysis in PFMS. The conversion is performed employing BEDTools [10] and samtools [11]. The application analyzes one chromosome at a time which implies extraction of the tags of the specified chromosome in a given data set. It is achieved by employment of FindPeaks split tool [3]. PFMS repeats the analysis process for all the chromosomes included in the dataset unless specified otherwise. Although the BED format is widely used, some of the included peak finders require the data set to be given in custom formats (e.g. Erange [1]). Therefore, PFMS internally converts the input data according to the required format of the peak finders.

### Data processing

Seven peak identification methods are currently integrated in PFMS. They are run separately to identify lists of putative peaks. The peaks are collected, normalized and converted to either BED or WIG, as selected by the user. The BED option is more convenient for additional downstream analysis, while WIG is better for visualization purposes since it retains information about the peak shapes.

#### BED option

PFMS selects an integrated list of significant peaks from the combined results by any of the following methods: voting mechanism, minFP or minFN. The first method does not use any normalization, while the other two use normalized scores of peaks obtained from the individual peak finders. The normalization is carried out using one of five different normalization methods described below.

#### WIG option

**Figure 1:**
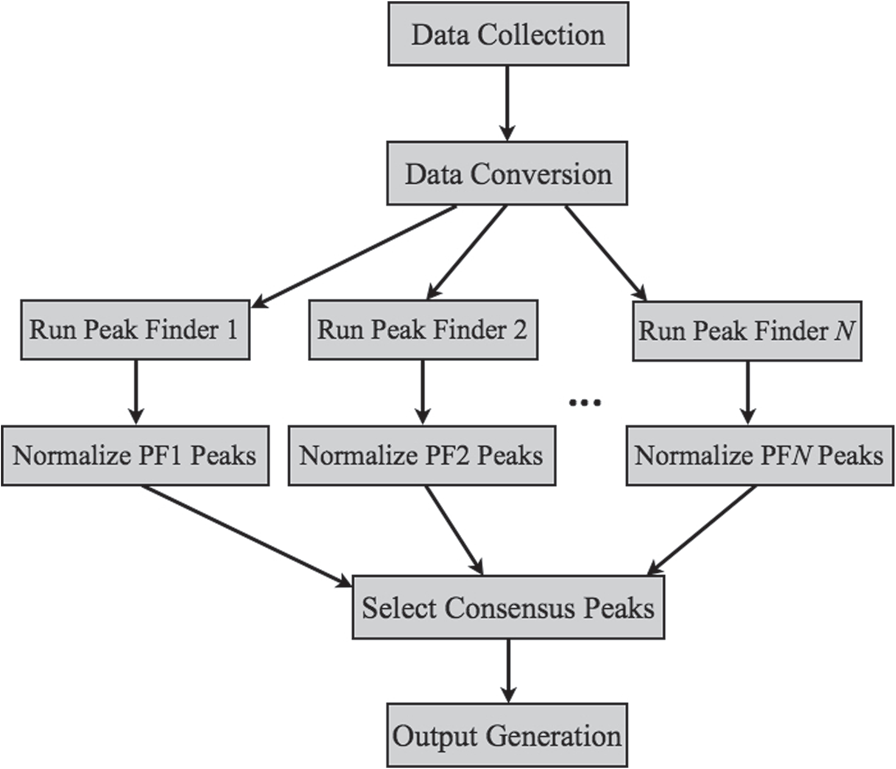
**PFMS block diagram.** The diagram of the algorithm of the PFMS. Peak finders 1, …, N are run independently, then selection of significant peaks may be done in three different ways - *minFP*, *minFN* and *Voting*. If *Voting* is selected then the normalization step is skipped. Normalization can be done in five different ways: *Normal*, *Naive Quantile*, *Average*, *Rank*, and *Top Rank*.

WIG files store information about the shape of the peaks. Firstly, peaks are divided into steps and any operation on the file is in fact done at the level of steps, not the whole peaks. Therefore PFMS normalizes the peak scores using the Average or Naive Quantile method as described below. Ranking the steps or normalizing them using Normal method does not make sense from the statistical point of view, thus these two methods have not been implemented for WIG option. Finally, PFMS integrates the overlapping peaks and sums up their scores. The results are easy to visualize using genome browsers, such as UCSC Genome Browser either in WIG or BED format. An overview of the implemented procedure is shown in Figure 1.

### Peak-score normalization

The peaks detected by the individual peak finders are usually scored by various enrichment measures. Due to different ranges of the scores, weighting of the selected peaks may be biased to the peaks ranked with higher scaled scores. To overcome this drawback, the output from each peak finder is normalized; five normalization methods are implemented.

#### Normal normalization

The program calculates the mean value and the standard deviation of the peaks scores identified by the selected peak finders. The peak scores are then transformed to have mean value of the *normal_shift* parameter and unit standard deviation. The remaining negative peak scores are set to 0, while they are kept in the down-stream analysis for voting. Any default value for the *normal_shift* parameter is set to 3 (1). The negative peak scores are considered as outliers since they are more than 3 (or the given *normal_shift* value) standard deviations from the mean value and can be set to 0 without any significant impact on the analysis. The fraction of such peaks for *normal_shift* of 3 is around 0.13%, provided the scores in the file have normal distribution. However, in real datasets, peak scores rarely exhibit normal distribution, and so few if any peak scores are set to 0. The scores are then multiplied by the maximum number of peaks taken from all peak finders and rounded up.

(1) $$\begin{array}{ll} \text{normalized\_peak\_score}= & \left[\frac{\text{peak\_score}-\bar{\text{peak\_score}}}{\text{sd}}\right)\\ & \left(+\text{normal\_shift}\;\right]*\text{max\_no\_of\_peaks} \end{array}$$

where: **normalized_peak_score-** score of the peak after Normal Normalization **peak_score-** original score of the peak $\bar{\text{peak\_score}}$**-** the mean score in the results of the currently normalized peak finder **sd-** standard deviation of scores in the results of the currently normalized peak finder **normal_shift-** the mean value of the scores after normalization **max_no_of_peaks-** maximal number of peaks returned by the peak finders

For example, five peaks with scores (2, 4, 4, 8, 12) were obtained from SISSR, while 10 peaks were obtained from MACS, which was the highest number of peaks from a single peak finder. The mean value was 6 and the standard deviation was 4. After applying Normal Normalization, the SISSR scores were normalized to (20, 25, 25, 35, 45). This normalization type may be used with BED option only.

#### Naive quantile normalization

The user defines which quantile is to be used for normalization (e.g. quantile 0.75.) The peak scores are sorted within the files with original scores from selected peak finders and the specified quantile is selected. Then, each peak score from the currently normalized peak finder is divided by the value of the selected quantile and, as in the previous method, multiplied by the maximum number of peaks in the results of the peak finders and rounded up (2).

(2) $$\begin{array}{ll} \text{normalized\_peak\_score}= & \frac{\text{peak\_score}}{\text{quantil}e_{n}}\\ & *\text{max\_number\_of\_peaks} \end{array}$$

where: **normalized_peak_score -** score of the peak after Naive Quantile Normalization **peak_score-** original score of the peak **quantile**_**n**_**-** the n-th quantile of the peak scores from the currently normalized peak finder **max_no_of_peaks-** maximal number of peaks returned by the peak finders

As an example, SISSR returned five peaks with scores (2, 4, 4, 8, 12), while 10 peaks were obtained from MACS, which was the highest number of peaks from a single peak finder. By using quantile 0.8, the peak scores of SISSR were normalized to (2.5, 5, 5, 10, 15), and rounded up to (3, 5, 5, 10, 15). Naive Quantile Normalization type may be used with the BED and WIG options.

#### Average normalization

This normalization is very similar to the Naive Quantile Normalization. The only difference is that instead of selecting a certain quantile, the mean value of all the scores is used. Average Normalization is performed according to 3.

(3) $$\begin{array}{ll} \text{normalized\_peak\_score}= & \frac{\text{peak\_score}}{\bar{\text{peak\_score}}}\\ & *\text{max\_number\_of\_peaks} \end{array}$$

where: **normalized_peak_score -** score of the peak after Average Normalization **peak_score -** original score of the peak $\bar{\text{peak\_score}}$**-** mean peak score in the currently normalized peak finder results **max_no_of_peaks-** maximal number of peaks returned by the peak finders

As an example, SISSR returned five scores (2, 4, 4, 8, 12), while 10 peaks were obtained from MACS, which was the highest number of peaks from a single peak finder. The mean value of SISSR peak scores, which was 6, was used to normalize the peak scores of SISSR to (3.33, 6.66, 6.66, 13.33, 20), after rounding (3, 7, 7, 13, 20). Average Normalization type may be used with the BED and WIG options.

#### Rank normalization

After sorting the lists of the peaks detected by the peak finders, the peaks are clustered using their scores, so that all peaks with the same score are in the same cluster. Next, each cluster gets a rank computed according to the following equation (4).

(4) $$\begin{array}{ll} \text{Cluster\_scor}e_{i}= & \left[\frac{\text{no\_of\_peak}s_{i}}{2}+\overset{i-1}{\underset{k=0}{\sum }}\text{no\_of\_peak}s_{k}\right]\\ & *\frac{\text{max\_no\_of\_peaks}}{\text{no\_of\_peaks\_from\_PF}} \end{array}$$

where: **Cluster_score**_**i**_**-** rank (score) for all peaks in the cluster *i***no_of_peaks**_**i**_**-** the number of peaks in cluster *i***max_no_of_peaks -** maximal number of peaks returned by the peak finders **no_of_peaks_from_PF -** number of peaks returned by the currently normalizing peak finder

For example, five peaks with scores (2, 4, 4, 8, 12) were obtained from SISSR, while MACS produced 10 peaks, which was the highest number of peaks obtained from a single peak finder. After applying the rank normalization method the SISSR peak scores were normalized to 1, 4, 4, 7, 9. Rank Normalization type may only be used with BED option.

#### Top rank normalization

In contrast to the previous normalization methods, Top Rank Normalization assumes that the methods that return fewer peaks also return a smaller fraction of False Positives. In this method, similarly to Rank Normalization, after sorting the lists of the peaks detected by the peak finders, the peaks are clustered using their scores, so that all peaks with the same score are in the same cluster. The clusters are sorted starting with the highest-scoring cluster and ending with the lowest-scoring one. Next, each cluster gets a rank computed according to the following equation (5).

(5) $$\begin{array}{ll} \text{Cluster\_scor}e_{i}= & \text{max\_no\_of\_peaks}\\ & -\left[\frac{\text{no\_of\_peak}s_{i}-1}{2}+\overset{i-1}{\underset{k=0}{\sum }}\text{no\_of\_peak}s_{k}\right] \end{array}$$

where: **Cluster_score**_**i**_**-** rank (score) for all peaks in the cluster *i***no_of_peaks**_**i**_**-** the number of peaks in cluster *i***max_no_of_peaks-** maximal number of peaks returned by the peak finders

For example, five peaks with scores (12, 8, 4, 4, 2) were obtained from SISSR, while MACS produced 10 peaks, which was the highest number of peaks obtained from a single peak finder. After applying Top Rank Normalization method the SISSR peak-scores were normalized to 10, 9, 7.5, 7.5, 6, and rounded up to 10, 9, 8, 8, 6. Top Rank Normalization may be used with BED option only.

### Result generation

#### BED option

Prior to peak-selection, overlapping regions of the peaks obtained from each peak finder are aggregated and weighted by summing up the normalized scores of the regions. Apart from the aggregated score, the number of votes for each region is kept. The number of votes is defined as the number of peak finders that called the region. Finally, the consensus peaks are selected based on one of the following methods.

##### In-degree Centrality Voting Mechanism

**Figure 2:**
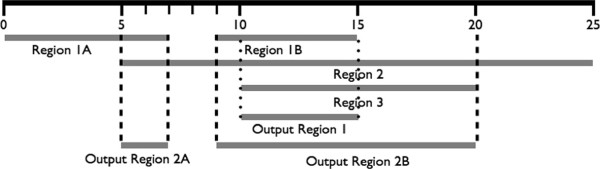
**PFMS BED output generation.** An example of PFMS output generation process for BED files in a short fragment of a genome. Output Region 1 is reported if the threshold is set to 3 and Output Region 2A and 2B are returned if the threshold is set to 2.

Is our simplest mechanism of peak selection. All the aggregated regions are considered as candidate sites. A putative binding site is reported if the number of votes for a region is higher than or equal to a predefined threshold (*min_rank*). Note, that in this method the scores generated by the peak finders are not utilized. For example, given a set of regions 1A: [0 - 7], 1B: [9 - 15], 2: [5 - 25], and 3: [10 - 20] generated by PF 1, PF1, PF2 and PF3, respectively, with the threshold value 2, the regions [5 - 7] and [9 - 20] are reported as binding sites. If the threshold was set to 3, the output region would be [10 - 15] (Figure 2).

##### minFP peak selection method

Attempts to minimize the number of false positive peaks, at the cost of losing some true positive peaks. In this method the user defines *min_rank*, which is the minimum number of votes that a region has to receive in order to be identified as a putative peak. We calculate *MaxP*, the highest score of a peak having fewer than *min_rank* votes. Each peak that has a score higher than *MaxP* is reported as a putative binding site.

##### minFN peak selection method

Attempts to minimize the number of false negative peaks, at the cost of including some false positive peaks. *min_rank* is defined as in *minFP*. *MinP* is the lowest score of the peak having *min_rank* or more votes. Each peak that has a score higher than *MinP* is reported as a putative binding site.

#### WIG option

With the WIG option, peaks are divided into steps, each characterized by a score. The number of scores and the step size determine the width of the peak. All step sizes are unified to the smallest value among all WIG files returned by individual peak finders. All identified peaks are collected and the overlapping regions integrated. The integrated regions are weighted with the normalized score of the overlapping peaks. Optionally, the highest weighted peaks may be selected from the list of the integrated regions by setting a cut-off value. The goal is to select the regions that are called by the majority of the peak finders and that have high scores. This leads to a set of enriched regions that are easy to visualize.

## Results and discussion

**Figure 3:**
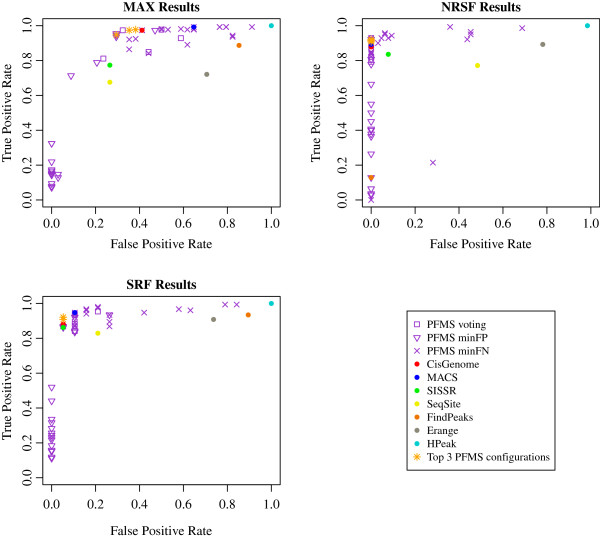
**PFMS versus single peak finders performance comparison.** Performance of the seven peak finders included in PFMS and PFMS itself with different settings. “PFMS voting”, “PFMS minFP” and “PFMS minFN” show results of PFMS with various combinations of peak finders, various minrank values but with *voting*, *minFP* and *minFN* options respectively. The orange “+” sign shows the results of the method we identified as the optimal one for finding peaks in the transcription factor ChIP-seq datasets. PFMS with *voting* option and *min_rank* parameter set to 2 using following peak finders: CisGenome, MACS, SISSR was the best choice. The purple points represent performance of PFMS with various parameters and various sets of peak finders included in the analysis.

Our method was evaluated against the benchmark datasets published by Rye et al. [12]. They analyzed ChIP-seq reads of three transcription factors (MAX, NRSF and SRF) using five different peak finders (MACS, SISSRs, PeakSeq, FindPeaks and QuEST). Furthermore, the authors visually inspected a number of detected peaks, classifying them as True Positive, False Positive or Ambiguous, providing an excellent resource for evaluation of peak finder performance. In our analysis we ran all seven peak finders included in PFMS on the three datasets incorporating control data with those peak finders that support experimental background measurements. PFMS with various combinations of parameters and peak finders was evaluated. The peaks obtained by the peak finders and by PFMS were intersected with the Rye’s results using BEDTools [10], keeping track of counts of True Positives and False Positives for each data set and parameter setting. The performance results of all runs are shown in Figure 3.

In order to clearly compare the performance of single peak finders and various configurations of PFMS three different measures were applied. Firstly, we calculated Euclidian distance of the points on the Figure 3 to point (0,1) for each of the investigated TFs. Secondly, for each configuration, we calculated *Average Euclidean Distance* for the three TFs results (see Additional file 1: Table S1). However, in the first measure the largest impact on the overall output have the results for the most 'difficult’ TF. A ranked-based approach was applied to compensate for this factor. We ordered increasingly the Euclidean distances separately for each TF. For each configuration we summed up the ranking position from all three TFs obtaining *Combined Ranking Score*. The third measure implemented in order to compare the performance of the peak finders and various configurations of PFMS was the *Average Normalized Distance*. To calculate this measure we applied studentization on the distances for each of the TF datasets. Then, for each peak finder and PFMS configuration we calculated mean value of the measure for the three datasets. For all three measures, the lower the score, the better configuration is.

In our validation analysis, the peak finders were ran with the default settings. However, tuning settings of the peak finders for the given datasets may improve the results. Configuration of the individual peak finders may be easily modified within PFMS (for detailed information see http://bioinf.icm.uu.se/~pfms/). CisGenome, MACS and SISSR were found to perform much better than the remaining four peak finders. Not surprisingly, PFMS performed best with only the three peak finders included. The top three choices for transcription factor dataset analysis utilize only these three peak finders with the *min_rank* parameter set to 2. We recommend either *voting* or *minFP* with Rank Normalization or Top Rank Normalization as these three configurations outperform each of the single peak finders as well as any other configuration of PFMS. Depending on the quality measure, one of the three configurations was the optimal one. However using any of the three quality measures the above mentioned configurations make the top three choices. As predicted, the *minFP* option returns results with only few False Positives but quite often misses True Positives. Apparently, this leads to a very high specificity and can be useful for applications such as selecting the strongest putative TF target genes for biological validation [13]. We recommend using the *minFP* option with CisGenome, MACS, SISSR and optionally with SeqSite.

In contrast, *minFN* has very high sensitivity, i.e. it returns most of the True Positives at the cost of including a considerable number of False Positives. This option also performs best with the four peak finders. The choice of normalization type did not seem to be crucial for the quality of the results when using *minFN* or *minFP*. Nevertheless, we do not recommend using the Normal Normalization unless one is certain that the peak scores obtained from all peak finders have a bell-shaped distribution. Otherwise, it is much safer to use Naive Quantile Normalization (e.g. quantile 0.75), Average or Rank Normalization. These methods do not require any assumptions about the distribution of the scores.

The configurations described in this section proved to be the best for transcription factor ChIP-seq datasets. Other types of ChIP-seq data such as histone modifications were not tested. Further investigation needs to be carried out and more validated datasets have to be provided to reveal the optimal settings for both PFMS itself as well as the individual peak finders. As an example, histone modifications ChIP-seq datasets are likely to have varying peak widths and shapes depending on the pattern of the modification (e.g. single or consecutive nucleosomes) and the density of the chromatin. Therefore, different approaches and options might be better for different cases.

PFMS can be used as a single interface for analyzing ChIP-seq datasets employing several peak finders simultaneously since users may choose a set of peak finders amongst the ones currently integrated in this application. In addition to the list of putative peaks identified by PFMS, the results of each peak finder may be stored in the output directory of PFMS.

## Conclusions

We present Peak Finder Metaserver - a novel tool for finding peaks in ChIP-seq data. The tool combines the results from various widely used methods and generates consensus results. We investigated seven peak finders and identified three that perform best for transcription factor ChIP-seq datasets, i.e. CisGenome, MACS and SISSR. Applying only these three peak finders and setting the *voting* peak selection method and the *minrank* parameter to 2. To the best of our knowledge this is the best method of finding peaks in transcription factor ChIP-seq datasets. Meta-Server approach proved to be successful and PFMS with the above mentioned settings generates results of a better quality than any of the individual peak finders. Different configurations of our tool can be optimal for different types of analyses, but identification of optimal settings requires other validated datasets.

## Availability and requirements

•**Project name:** Peak Finder MetaServer

•**Project homepage:**http://bioinf.icm.uu.se/~pfms/

•**Operating system(s):** Linux, MacOS

•**Programming language:** python

•**Other requirements:** Python 2.6 or higher, GCC compiler, Perl, JRE 1.6

•**License:** GNU

•**Any restrictions to use by non-academics:** none

## Abbreviations

ChIP-seq: Chromatin immunoprecipitation sequencing; PFMS: Peak Finder Metaserver; TF: Transcription factor.

## Competing interests

The authors declare that they have no competing interests.

## Authors’ contributions

MK - Created the first version of PFMS with three peak finders (Erange, HPeak and MACS), implemented all the normalization and cut-off point evaluation methods, took part in testing the method, created all the figures and wrote the first version of the manuscript. HU - Made further developments of PFMS, added four peak finders (SISSR, SeqSite, CisGenome and FindPeaks), and integrated all peak finders. Converted the method to a multithread application, took part in testing the PFMS, took part in writing of the manuscript. SE - Conceived the system and took part in the writing of the manuscript. JK - Supervised the work and took part in the writing of the manuscript. All authors read and approved the final manuscript.

## Supplementary Material

Additional file 1 **Table S1.** An.xls file containing Table S1. Can be viewed with Microsoft Excel or similar software. Table S1 - The comparison of the results obtained from individual peak finders and various PFMS configurations. The Table contains results of the three quality measures for individual peak finders and various PFMS configurations. 'Distance’ means the Euclidian distance of a point from (0,1). 'Ranking’ corresponds to the Ranking quality measure described above. 'Normalized Distance’ is the Euclidean distance after studentization within the results for a certain TF dataset. The three last columns contain measures combined for all three TF datasets. The user may compare the performance of each peak finder and PFMS configuration by sorting appropriate columns. Click here for file
